# Observational cohort study protocol: neutrophil function and energetics in adults with pneumonia and sepsis – Pneumonia Metabolism in Ageing (PUMA)

**DOI:** 10.1136/bmjresp-2023-001806

**Published:** 2023-10-12

**Authors:** Frances S Grudzinska, Aduragbemi A Faniyi, Aaron Scott, Elizabeth Sapey, David R Thickett

**Affiliations:** 1 Birmingham Acute Care Research, University of Birmingham College of Medical and Dental Sciences, Birmingham, UK; 2 PIONEER Data Hub, University of Birmingham College of Medical and Dental Sciences, Birmingham, UK

**Keywords:** Pneumonia, Neutrophil Biology, Bacterial Infection, Innate Immunity, Respiratory Infection

## Abstract

**Introduction:**

Community-acquired pneumonia has high mortality and is associated with significant healthcare costs. In older adults with community-acquired pneumonia neutrophil dysfunction has been identified and is associated with poor outcomes for patients. Immunometabolism is a rapidly developing field which links immune cell function to metabolism. This study aims to explore neutrophil metabolism in community-acquired pneumonia.

**Methods and analysis:**

Pneumonia Metabolism in Ageing study is a prospective observational study recruiting older adults hospitalised with community-acquired pneumonia to examine neutrophil function and metabolic status. Controls will be older adults with no acute illness. The primary endpoint is neutrophil chemotaxis.

**Ethics and dissemination:**

The study has ethical approval from the Research Ethics Committee Wales, reference 19/WA/0299. This study involves participants who may lack the capacity to consent to research involvement, in this situation, personal or professional assent will be sought. The results from this study will be submitted for publication in peer-reviewed journals and disseminated at local and international conferences.

**WHAT IS ALREADY KNOWN ON THIS TOPIC**

- Neutrophils from older adults with community-acquired pneumonia demonstrate altered effector functions compared with younger adults. However, the mechanism underlying the broad dysfunction seen remains unclear.

**WHAT THIS STUDY ADDS**

- This study investigates neutrophil metabolism, a novel aspect of neutrophil biology in pneumonia and sepsis.

**HOW THIS STUDY MIGHT AFFECT RESEARCH, PRACTICE OR POLICY**

- This study has the potential to lead to a new treatment paradigm in community-acquired pneumonia; targeting the host response alongside antimicrobial therapies.

## Introduction

Community-acquired pneumonia (CAP) is a leading cause of hospitalisation and has high mortality in older adults.^1^ Mortality is greatest in those with sepsis due to CAP (CAP+S), with survivors experiencing increased mortality for a decade following discharge from hospital.^2^ The long-term burden of CAP includes cognitive dysfunction, reduced mobility and increased care needs. Readmission following hospitalisation for CAP is common, occurring in 11% of older adults.^3^

CAP occurs most commonly in adults aged over 65 years, and older adults are more likely to experience severe CAP and CAP+S.^4^ As the global population is ageing the prevalence of CAP is likely to rise.^5^

Between 22% and 42% of all CAP episodes require hospitalisation,^6^ and this places a significant burden on health services. In 2019, hospital treatment for CAP cost the National Health Service £731 million, with costs significantly higher for the treatment of older adults.^7^

There have been no significant advances in the management of CAP since the advent of antibiotic therapy.

Recovery from CAP requires a functional and coordinated immune response. In CAP, neutrophils are the predominant responding immune cell to infection and the resulting tissue damage. Neutrophils migrate to sites of inflammation and clear infection through bactericidal effector functions including phagocytosis and the generation of neutrophil extracellular traps and reactive oxygen species (RoS).^8^ These functions can be encompassed by cellular phenotype; the manifestation of cellular processes at gene and protein levels which result in changes in a cell’s function, morphology and surface markers.^9^

In young adults, CAP is associated with a rapid upregulation of effector functions until the infection is resolved.^10 11^ However, even in health neutrophil functions in older adults appear depressed,^12^ this is particularly true in frail older adults^13^ and data suggest that CAP exacerbates this, with further reductions in neutrophil functions leading to a state of immunoparesis which has been demonstrated for up to 6 weeks following CAP.^12 14–16^ Differences in neutrophil cellular phenotype have been identified in sepsis^17^ and disease states such as chronic obstructive pulmonary disease,^18^ however, there is no literature examining changes in surface marker expression combined with functional assessment in CAP.

This broad impact on effector function suggests that a central cellular mechanism is involved, such as metabolism. Immunometabolism is defined as the role of cellular metabolism in the regulation of immune function^19^ and there is increasing interest in exploring if therapeutically targeting immunometabolism may optimise immune responses. For example, inhibiting glycolysis in myeloid cells using murine models is associated with an improved recovery from sepsis.^20^

Neutrophil metabolism is poorly understood, especially in disease states but neutrophils appear to rely on glycolysis for energy generation.^21^ The effector functions required for a coordinated and effective response to infection are highly energy-demanding processes. Modification of glycolysis in neutrophils may offer a novel therapeutic strategy for CAP. Potential targets include glucose transporters, key enzymes required for glycolysis (hexokinase, phosphofructokinase, pyruvate kinase and lactate dehydrogenase) and lactate transporters/receptors. Evidence to date confirms alterations in these key enzymes during sepsis.^22^

This manuscript describes a protocol for the assessment of neutrophil function, phenotype and immunometabolism in older adults with CAP, compared with an age, frailty and morbidity-matched cohort in the Pneumonia Metabolism in Ageing (PUMA) study.

## Study aims

### Research hypothesis and aims

We hypothesise that depressed neutrophil function seen in older adults with CAP is due to alterations in neutrophil immunometabolism, and that this alteration is modifiable in vitro by modulating glycolysis.

**Figure 1:**
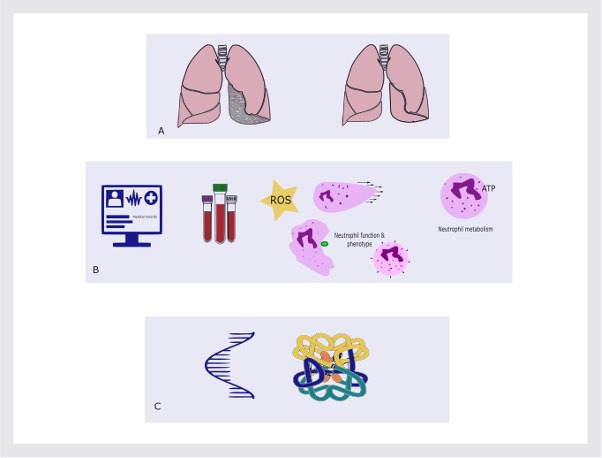
Schematic overview of study design and outcomes. (A) Older adults with and without community-acquired pneumonia (CAP) will be recruited, (B) we will collect a detailed medical history, record complications and outcomes of CAP, participants will donate whole blood used to isolate neutrophils. Isolated neutrophils will be functionally assessed (oxidative burst and migration), phenotype as assessed by cell surface markers and metabolic status will be measured. (C) Changes seen in function and metabolism will be assessed at RNA and protein levels. For CAP participants, further visits will be repeated at days 3–5 and day 42 following admission. Created with BioRender.com

This study aims to functionally phenotype neutrophils from participants with CAP alongside age and frailty-matched cohort while characterising the metabolism of these cells. Figure 1 provides an overview of the study design and outcomes collected. Primary and secondary aims are outlined in table 1.

**Table 1:** Primary and secondary aims of the PUMA study

Primary aims:	Sequentially assess community-acquired pneumonia-related change in neutrophil phenotype Characterise neutrophil metabolism in cases and controls
Secondary aims:	Migration Oxidative burst Phenotype as defined by cell surface markers Transcriptomics Determine if neutrophil function can be prevented or restored by modulating neutrophil metabolism using targets involved in glycolysis

PUMA, Pneumonia Metabolism in Ageing.

## Methods

### Study design

PUMA is an observational cohort study recruiting adult participants with CAP as defined by the British Thoracic Society.^23^ This includes symptoms and signs consistent with an acute lower respiratory tract infection associated with radiographic shadowing for which there is no other explanation. Signs and symptoms include three or more of the following: cough, sputum production, breathlessness, pleuritic chest pain, haemoptysis, fever, headache and signs consistent with pneumonia on chest auscultation. Participants with and without sepsis will be recruited. Sepsis is defined as a quick Sequential Organ Failure Assessment score score ≥2.^24^

The control cohort will be matched as much as possible for age and frailty with no recent acute illness. Matching for frailty will be achieved using clinical frailty scale. Participants will be recruited between March 2020 and November 2024; however, there have been significant periods of disruption relating to the pandemic and other factors. Recruitment is ongoing.

Full inclusion and exclusion criteria for each cohort are in table 2. We have chosen to exclude patients with common conditions or medications known to significantly impact neutrophil function and or metabolism such as chronic obstructive pulmonary disease and haematological malignancy.^25 26^

**Table 2:** Inclusion and exclusion criteria

Inclusion criteria	Exclusion criteria
CAP cohort	
Community-acquired pneumonia±sepsis admitted to hospital Age ≥65 years	Asthma Chronic obstructive pulmonary disease Metastatic malignancy Haematological malignancy Participants already enrolled in a study of novel/unlicensed treatment Immunosuppressive treatment in the preceding 12 weeks Known immunodeficiency syndrome Treatment withdrawal imminent Current infection with COVID-19
Control cohort	
Age ≥65 years	As above plus: Acute illness in preceding 12 weeks Unable to give informed consent to enrolment

CAP, community-acquired pneumonia.

The study will be conducted in a single site, at the Queen Elizabeth Hospital, Birmingham, UK (QEHB). The QEHB is a 1200-bed tertiary and acute hospital in an urban area.

### Sampling and sample size calculation

CAP cases will be recruited from inpatient wards at QEHB and are expected to remain admitted for ≥24 hours. Controls will be recruited from a range of outpatient clinics at QEHB to enable matching of age, frailty and comorbidity where possible.

The primary outcome measure for sample size calculation is based on a reduction in chemotaxis in patients with CAP compared with age-matched controls. Pilot data indicate a 62% reduction in chemotaxis in CAP cases with SD 0.079 for CAP and 0.135 for controls. To detect a difference in chemotaxis with p<0.005 and 95% power, we will need to recruit eight CAP and eight controls. In the completed SNOOPI study, a 30-day mortality was 16%, and study withdrawal was 6%.^11^ We estimate we need to recruit two CAP participants to account for drop-outs. Total sample size: 10 CAP and 8 controls. This limited sample size is not anticipated to inform relationships between clinical outcomes and neutrophil function but is powered to detect a biological endpoint (difference in neutrophil chemotaxis between CAP and controls).

### Study visit procedure

#### Participant identification, consent and follow-up

**Figure 2:**
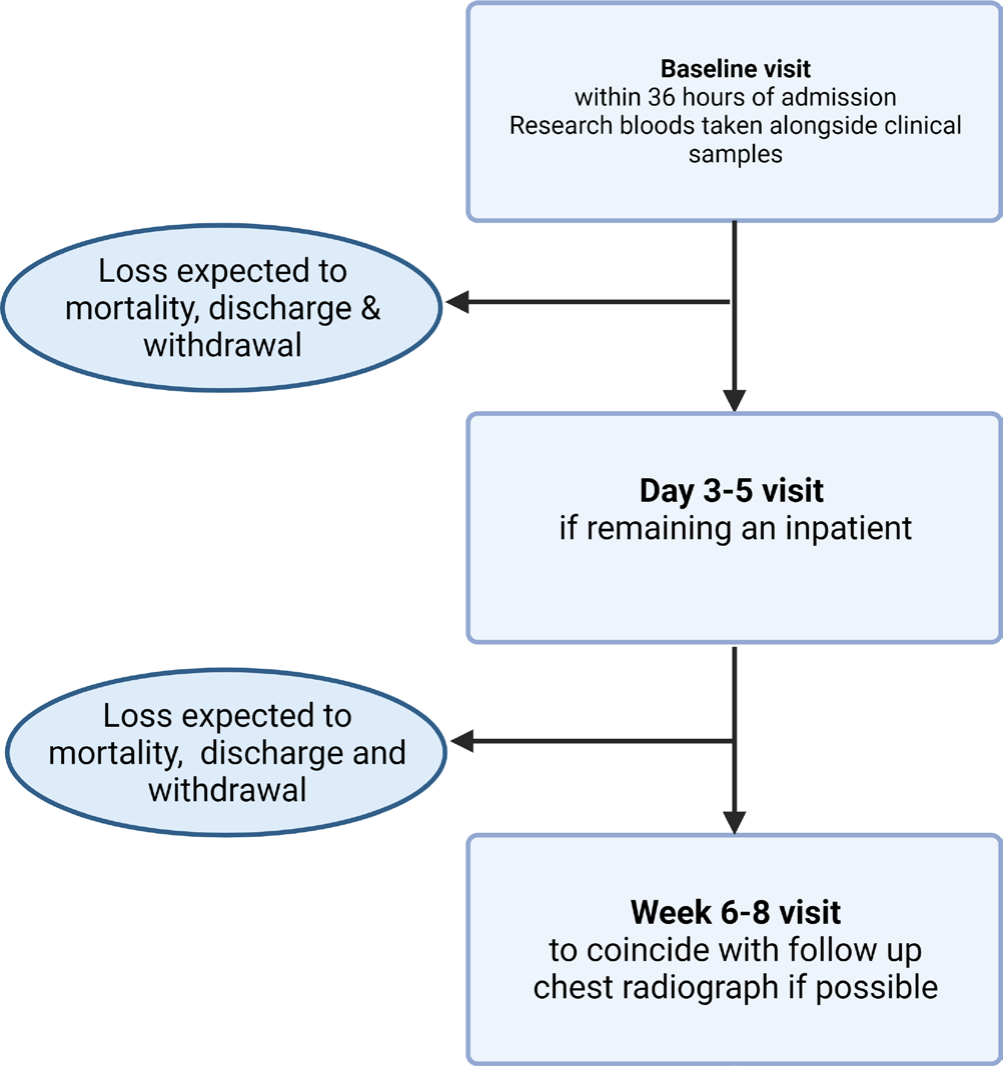
Study procedures for CAP participants. CAP participants will be recruited for baseline visit within 24 hours of admission, at this visit research blood samples will be taken alongside clinical bloods and demographic data collected. If remaining an in-patient at 3–5 days a further research blood sample will be collected. A visit at 6–8 weeks following admission will be arranged to coincide with follow-up imaging if requested by clinical team. Created with BioRender.com. CAP, community-acquired pneumonia.

Where possible CAP participants will be recruited within 36 hours of hospital admission, then follow-up visits between day three and five (if remaining an inpatient) and then 6–8 weeks following admission, where possible the 6–8 weeks visit will be timed to coincide with follow-up chest radiograph when requested by the clinical team. Drop-outs to follow-up are expected due to death, discharge prior to days 3–5, withdrawal and unwillingness to return to hospital for 6–8 weeks visit. Study procedures are shown in figure 2. CAP frequently causes delirium, and dementia is a common comorbidity of older adults with CAP, therefore, CAP participants who lack the ability to consent to enrolment are not excluded. In the case of impaired capacity to consent to study enrolment a personal consultee opinion will be sought in the first instance, if no personal consultee is available an independent professional consultee’s opinion will be sought. Retrospective consent will be sought in these cases.

Control participants will be recruited at a single time point only.

#### Data collection

**Figure 3:**
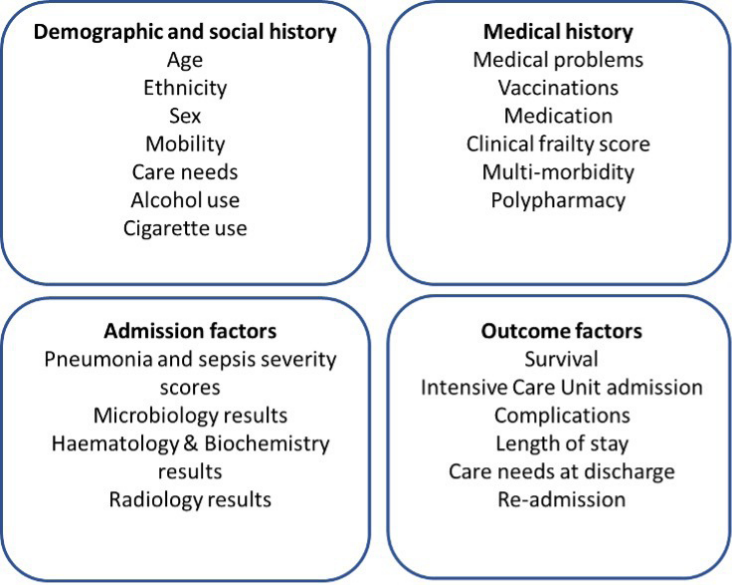
Clinical data collected at study visits: at study visits, clinical information will be recorded in the following domains: demographic and social history, medical history, admission factors and outcome factors.

At each study visit participants will undergo a detailed clinical history to ascertain factors which may influence neutrophil function, and outcome of CAP, as described in figure 3. We will also record information related to the hospital admission such as treatment strategies that may influence outcomes. Traditionally studies of CAP have considered binary outcomes such as survived/died, however, our public and patient involvement and engagement work demonstrated that a more nuanced approach was preferred. Therefore, we will collect information on postdischarge mobility, care needs and readmissions to hospital. Due to the limited size of the planned study, the comparisons using this data are intended to be exploratory and ensure the CAP and control cohorts are well matched.

#### Blood sampling

For CAP participants, blood samples for research will be taken alongside clinically indicated blood samples to reduce number of venepuncture episodes for both CAP and control participants. Maximum of 45 mL blood will be taken at each study visit for research purposes.

#### Biological samples

Blood samples will predominantly be used in neutrophil isolation, processing and downstream use is described in table 3.

**Table 3:** Biological sample processing

Vacutainer type	Volume (mL)	Purpose	Processing	Storage
Lithium Heparin	24	Neutrophil isolation	Described in text	N/A
Lithium Heparin	6	Plasma samples	Sedimentation at room temperature for 30 min followed by centrifugation at 560 rpm for 10 min.	500 µL aliquots at −80°C
EDTA	4	Plasma samples	As above	500 µL aliquots at −80°C
Plain	6	Serum sample	As above	500 µL aliquots at −80°C

N/A, not available.

#### Neutrophil isolation and functional assays

Isolated neutrophils will be prepared by discontinuous Percoll density centrifugation.^18^ Briefly, whole blood is gently mixed with 2% dextran in 6:1 ratio to enable sedimentation of red blood cells. Discontinuous Percoll solutions are prepared by diluting in either 1.54M NaCl or 0.154M NaCl to generate 90% and 56% solutions, respectively. The buffy coat is then layered over the discontinuous Percoll solution. The gradient is centrifuged at 470 g for 20 min at room temperature with no break or acceleration. The neutrophil layer is harvested and washed in phosphate buffered saline (PBS) to form a pellet. The neutrophils are resuspended in RPMI 1640 for downstream use. Neutrophils are typically >97% pure and >95% viable as assessed annexin/propidium iodide staining. Due to limitations in cell number, not all experimental assays will be carried out for each participant.

Neutrophil chemotaxis is assessed to a chemoattractant (interlukin-8) using a modified Insall chamber as described in.^26^ Use of the Insall chamber with time lapse filming using video microscopy allows for quantification of speed and directionality of individual neutrophils. To analyse the time lapse filming 10 neutrophils are selected at random and their movement tracked using FIJI Image J. These tracking data are then used to calculate speed, velocity, persistence and overall accuracy. Neutrophils migrate using proteinases to navigate extracellular matrices; therefore, speed and accuracy are important in assessing migratory parameters.^26^

Neutrophil lysates are prepared for RNA extraction and protein lysate for Western blotting and stored at −80°C. RNA will be used in RNA sequencing, targets identified involved in metabolism will be confirmed at the protein level.

Neutrophil oxidative burst is assessed using the Seahorse Analyzer (Agilent Technologies). In brief, neutrophils are treated with inhibitors of mitochondrial respiration (rotenone/anti-mycin A) followed by phorbol 12-myristate 13-acetate (PMA) treatment to generate RoS.^27^ Oxygen consumption is then measured to assess oxidative burst from the neutrophils over a time course. This method allows comparison of overall oxygen uptake and time taken to reach maximal oxygen uptake.

#### Neutrophil cell surface marker expression

Neutrophil phenotype will be assessed by staining isolated neutrophils with antibodies that bind to extracellular surface markers. Antibodies to detect cluster of differentiation (CD) 11b, CD66b, CD10, CXC chemokine receptor (CXCR) 2 and CXCR4 are mixed as one cocktail, while antibodies that bind CD16, CD11c, CD62L, CD54 and programmed death-ligand (PD-L) 1 are mixed as a second cocktail. The antibody cocktails as well as isotype controls are added to neutrophils and incubated on ice for 30 min in the dark. Unstained controls are also included in the incubation. After incubation, neutrophils are washed with 1% bovine serum albumin in PBS (FACS buffer) and centrifuged at 300 g for 5 min. The neutrophils are resuspended in FACS buffer and analysed using a MACSQuant 10 flow cytometer (Miltenyl Biotec). Data are analysed using FlowJo software. The analysis of the expression of surface receptors will allow for comparison of neutrophil phenotype in terms of their activation, maturity, senescence, reverse migration, respiratory burst and immunosuppression.

#### Glycolytic rate

Neutrophil metabolism will be assessed using the Seahorse Analyzer (Agilent Technologies) to measure extracelllar acidification rate (ECAR) of media as a surrogate of lactate efflux.^27^ The assay involves use of both glycolytic and mitochondrial inhibitors to interrogate metabolic pathways. Freshly isolated neutrophils are seeded on a cell plate, ECAR is measured over time with injection of PMA (a potent stimulator of neutrophils) after measurement of baseline glycolysis, a subsequent injection of oligomycin inhibits mitochondrial activity to confirm that all measured activity is glycolytic in origin. Lastly, 2-deoxyglucose, an inhibitor of glycolysis, is added to confirm all metabolic activity is shutdown. Baseline glycolysis, maximal glycolysis and induced glycolysis can be measured using this assay.

#### Transcriptomics

Transcriptomic analysis will be carried out in collaboration with Genomics department at University of Birmingham. Total RNA will be extracted using the RNAqueous Micro Total RNA Isolation Kit (to include an on-column DNase treatment). RNA quality will be measured using the Agilent 2200 Tapestation. The polyA+mRNA fractionation will be isolated and cDNA libraries prepared using the QuantSeq 3‘mRNA-Seq Library Prep Kit. Samples will then be subjected to 75 bp, single-end sequencing using Illumina 2500 sequencing machine. Paired end reads will be aligned to the human reference genome (hg38) using STAR and differential gene expression will be determined using DESeq2.

### Public and patient involvement and engagement

Patient involvement led to the following changes in the research protocol; where possible the 6-week follow-up visit would occur either as a home visit, or when the participant returned to the hospital for follow-up chest radiographs as commonly occurs following treatment for CAP. In addition, following discussion with patients we expanded information collected on follow-up to include care needs, readmission and mobility changes. Finally, we included a follow-up letter at the end of the study to all participants to thank them for their involvement and update them on the study’s findings. All study documents have been reviewed by patient representatives.

### Statistical analysis

Data will be analysed using SPSS for Windows V.26.0, GraphPad Prism V.9.0 or R V.4.2 or later. For continuously distributed data, differences between groups will be tested using independent sample t-tests with transformations of variables to normality if appropriate or non-parametric equivalents. χ^2^ tests (or Fisher’s exact tests) will be used for categorical variables. Any comparisons of clinical parameters such as impact of CAP severity will be considered exploratory.

The results of this study will be submitted to peer-reviewed journals for publication and will be presented at local and national conferences. We will adhere to Enhancing the QUAlity and Transparency Of health Research (EQUATOR) guidelines for observational studies and follow International Committee of Medical Journal Editors (ICMJE) best practice in generation of the study manuscript. No participants will be identified in publications that result from this study.

### Dissemination

Local monitoring of the study will include the following: confirmation of informed consent or assent, source data verification, backup and validation of data input. A random sample (10%) of case report forms will be checked to ensure accurate data input.

We do not anticipate any safety concerns during this study. Any adverse events will be immediately reported to the chief investigator, and they will assess causality and seriousness.

## Data Availability

Data sharing not applicable as no datasets generated and/or analysed for this study.

## References

[1] Welte T, Köhnlein T. Global and local epidemiology of community-acquired pneumonia: the experience of the CAPNETZ network. Semin Respir Crit Care Med 2009;30:127–35. 10.1055/s-0029-1202941 19296412

[2] Martin GS, Mannino DM, Moss M. The effect of age on the development and outcome of adult sepsis. Crit Care Med 2006;34:15–21. 10.1097/01.ccm.0000194535.82812.ba 16374151

[3] Toledo D, Soldevila N, Torner N, et al. Factors associated with 30-day readmission after hospitalisation for community-acquired pneumonia in older patients: a cross-sectional study in seven Spanish regions. BMJ Open 2018;8:e020243. 10.1136/bmjopen-2017-020243 PMC5884368 29602852

[4] Ewig S, Birkner N, Strauss R, et al. New perspectives on community-acquired pneumonia in 388 406 patients. results from a nationwide mandatory performance measurement programme in healthcare quality. Thorax 2009;64:1062–9. 10.1136/thx.2008.109785 19454409 PMC2782114

[5] United Nations. World population prospects. 2017. Available: https://esa.un.org/unpd/wpp/

[6] Guest JF, Morris A. Community-acquired pneumonia: the annual cost to the national health service in the UK. Eur Respir J 1997;10:1530–4. 10.1183/09031936.97.10071530 9230242

[7] Campling J, Wright HF, Hall GC, et al. Hospitalization costs of adult community-acquired pneumonia in England. J Med Econ 2022;25:912–8. 10.1080/13696998.2022.2090734 35726515

[8] Amulic B, Cazalet C, Hayes GL, et al. Neutrophil function: from mechanisms to disease. Annu Rev Immunol 2012;30:459–89. 10.1146/annurev-immunol-020711-074942 22224774

[9] Sul J-Y, Wu CK, Zeng F, et al. Transcriptome transfer produces a predictable cellular phenotype. Proc Natl Acad Sci U S A 2009;106:7624–9. 10.1073/pnas.0902161106 19380745 PMC2670883

[10] Patel JM, Sapey E, Parekh D, et al. Sepsis induces a dysregulated neutrophil phenotype that is associated with increased mortality. Mediators Inflamm 2018;2018:4065362. 10.1155/2018/4065362 29849488 PMC5925119

[11] Sapey E, Patel JM, Greenwood H, et al. Simvastatin improves neutrophil function and clinical outcomes in pneumonia a pilot randomized controlled clinical trial. Am J Respir Crit Care Med 2019;200:1282–93. 10.1164/rccm.201812-2328OC 31206313 PMC6857486

[12] Sapey E, Greenwood H, Walton G, et al. Phosphoinositide 3-kinase inhibition restores neutrophil accuracy in the elderly: toward targeted treatments for Immunosenescence. Blood 2014;123:239–48. 10.1182/blood-2013-08-519520 24191150 PMC3888290

[13] Wilson D, Drew W, Jasper A, et al. Frailty is associated with neutrophil dysfunction which is correctable with phosphoinositol-3-kinase inhibitors. J Gerontol A Biol Sci Med Sci 2020;75:2320–5. 10.1093/gerona/glaa216 32877922 PMC7662170

[14] Grudzinska FS, Brodlie M, Scholefield BR, et al. Neutrophils in community-acquired pneumonia: parallels in dysfunction at the extremes of age. Thorax 2020;75:164–71. 10.1136/thoraxjnl-2018-212826 31732687 PMC7029227

[15] Ebrahimi F, Giaglis S, Hahn S, et al. Markers of neutrophil extracellular traps predict adverse outcome in community-acquired pneumonia: secondary analysis of a randomised controlled trial. Eur Respir J 2018;51:1701389. 10.1183/13993003.01389-2017 29519921

[16] Sapey E, Patel JM, Greenwood HL, et al. Pulmonary infections in the elderly lead to impaired neutrophil targeting, which is improved by simvastatin. Am J Respir Crit Care Med 2017;196:1325–36. 10.1164/rccm.201704-0814OC 28657793 PMC5694832

[17] Lewis SM, Treacher DF, Edgeworth J, et al. Expression of CD11C and EMR2 on neutrophils: potential diagnostic biomarkers for sepsis and systemic inflammation. Clin Exp Immunol 2015;182:184–94. 10.1111/cei.12679 26153037 PMC4608508

[18] Hughes MJ, McGettrick HM, Sapey E. Importance of validating antibody panels: anti-PD-L1 clone binds AF700 Fluorophore. J Immunol Methods 2020;483:112795. 10.1016/j.jim.2020.112795 32464141 PMC7378575

[19] Bird L. Getting enough energy for immunity. Nat Rev Immunol 2019;19:269. 10.1038/s41577-019-0159-y 30940933

[20] Zheng Z, Ma H, Zhang X, et al. Enhanced glycolytic metabolism contributes to cardiac dysfunction in polymicrobial sepsis. J Infect Dis 2017;215:1396–406. 10.1093/infdis/jix138 28368517 PMC5451607

[21] Borregaard N, Herlin T. Energy metabolism of human neutrophils during phagocytosis. J Clin Invest 1982;70:550–7. 10.1172/jci110647 7107894 PMC370256

[22] Nalos M, Parnell G, Robergs R, et al. Transcriptional reprogramming of metabolic pathways in critically ill patients. Intensive Care Med Exp 2016;4:21. 10.1186/s40635-016-0094-1 27387528 PMC4936987

[23] Lim WS, Baudouin SV, George RC, et al. BTS guidelines for the management of community acquired pneumonia in adults: update 2009. Thorax 2009;64 Suppl 3:iii1–55. 10.1136/thx.2009.121434 19783532

[24] Seymour CW, Liu VX, Iwashyna TJ, et al. Assessment of clinical criteria for sepsis: for the third International consensus definitions for sepsis and septic shock (Sepsis-3). JAMA 2016;315:762–74. 10.1001/jama.2016.0288 26903335 PMC5433435

[25] Sadiku P, Willson JA, Ryan EM, et al. Neutrophils fuel effective immune responses through gluconeogenesis and glycogenesis. Cell Metab 2021;33:411–23. 10.1016/j.cmet.2020.11.016 33306983 PMC7863914

[26] Sapey E, Stockley JA, Greenwood H, et al. Behavioral and structural differences in migrating peripheral neutrophils from patients with chronic obstructive pulmonary disease. Am J Respir Crit Care Med 2011;183:1176–86. 10.1164/rccm.201008-1285OC 21257786

[27] Grudzinska FS, Jasper A, Sapey E, et al. Real-time assessment of neutrophil metabolism and oxidative burst using extracellular flux analysis. Front Immunol 2023;14:1083072. 10.3389/fimmu.2023.1083072 37180154 PMC10166867

